# Evaluation of the Accuracy of Cr and BUN Using the ABL90 FLEX PLUS Blood Gas Analyzer and the Equivalence of Candidate Specimens for Assessment of Renal Function

**DOI:** 10.3390/jcm12051940

**Published:** 2023-03-01

**Authors:** Ha-Jin Lim, Seung-Yeob Lee, Hyun-Jung Choi

**Affiliations:** 1 Department of Laboratory Medicine, Chonnam National University Medical School and Hospital, Gwangju 61469, Republic of Korea; 2 Department of Laboratory Medicine, Jeonbuk National University Medical School and Hospital, Jeonju 54907, Republic of Korea; 3 Research Institute of Clinical Medicine of Jeonbuk National University-Biomedical Research Institute of Jeonbuk National University Hospital, Jeonju 54907, Republic of Korea

**Keywords:** kidney disease, blood urea nitrogen, creatinine

## Abstract

Background: The ABL90 FLEX PLUS (Radiometer) is a blood gas analyzer that also provides creatinine (Cr) and blood urea nitrogen (BUN) results. We assessed the accuracy of the ABL90 FLEX PLUS to measure Cr and BUN and find suitable candidate specimens against primary specimens (heparinized whole-blood (H-WB)). Methods: Paired H-WB, serum, and sodium-citrated whole-blood (C-WB) samples (105) were collected. The Cr and BUN levels in the H-WB using the ABL90 FLEX PLUS were compared with those of the serum using four automated chemistry analyzers. The suitability of the candidate specimens was assessed at each medical decision level according to the CLSI guideline EP35-ED1. Results: The respective mean differences of the ABL90 FLEX PLUS for the Cr and BUN were below −0.10 and −3.51 mg/dL compared to the other analyzers. The systematic differences between the serum and the H-WB at the low, medium, and high medical decision levels were all 0% for Cr, but those of the C-WB were −12.96%, −11.81%, and −11.30%, respectively. Regarding imprecision, the SD_serum_/SD_H-WB_ ratios at each level were 0.14, 1.41, and 0.68, whereas the SD_C-WB_/SD_H-WB_ ratios were 0.35, 2.00, and 0.73, respectively. Conclusions: The ABL90 FLEX PLUS provided Cr and BUN results comparable with the four widely used analyzers. Among the candidates, the serum was suitable for Cr testing using the ABL90 FLEX PLUS, while the C-WB did not satisfy the acceptance criteria.

## 1. Introduction

Creatinine (Cr) and blood urea nitrogen (BUN) are two commonly measured parameters used to assess kidney function in clinical laboratories. The most frequently used devices for measuring Cr and BUN levels are automated chemistry analyzers, and point-of-care (POC) testing devices, including blood gas analyzers. Automated chemistry analyzers use chemical reactions to measure the concentration of Cr and BUN in blood samples. These analyzers are commonly found in clinical laboratories in hospitals and can analyze a wide range of other blood markers in addition to Cr and BUN. Beckman Coulter, Hitachi, Roche, and Siemens are widely recognized manufacturers of automated chemistry analyzers used in clinical laboratories [1].

The ABL90 FLEX PLUS analyzer (Radiometer, Copenhagen, Denmark) is primarily designed to measure blood gas levels but can also measure Cr and BUN levels, as well as other blood markers such as electrolytes, glucose, and lactate on heparinized (H-WB) [2,3,4]. It is easy to use and provides fast results, with built-in quality control features to ensure accuracy and reliability [5]. Point-of-care (POC) testing with the ABL FLEX PLUS for kidney function is a useful method for providing rapid results, particularly in emergency departments, acute medical units, or critical care settings where there is a need to make immediate decisions regarding treatment [6]. Ensuring patient safety before the administration of contrast media is particularly important, as highlighted by several studies [7,8,9,10,11]. However, the literature reveals both disparities in the clinical concordance with the central laboratory and in the clinical utility of POC in clinical practice, and therefore, its adoption has been limited [6,12]. These inconsistencies in Cr and BUN results have been caused by different sample types and measuring methods or analyzers [13,14], which may have largely affected the uncertainty of the estimated glomerular filtration rate (eGFR) at the medical decision level [15]. Therefore, providing precise and continuous Cr and BUN data is important for clinicians to determine the delta value for making medical decisions in individual patients even though the measurement equipment is different or changed. In addition, it is necessary and important to know the equivalence between different specimen types for Cr and BUN tests in order to respond quickly to various clinical situations [4,5,6,7,8,9,10]. However, there have been no comparative studies of Cr and BUN testing between two or more automated chemistry analyzers simultaneously with the ABL90 FLEX PLUS.

To address the unmet need for the evaluation of the ABL90 FLEX PLUS, this study evaluates the accuracy of Cr and BUN testing of the ABL90 FLEX PLUS by comparing these measurements to four automated chemistry analyzers widely used in clinical laboratories. Furthermore, the expandability of the sample selection was explored according to the up-to-date Clinical and Laboratory Standards Institute (CLSI) guideline EP35-ED1 for the first time by confirming the suitability of an alternative candidate specimen for Cr and BUN measurement using the ABL90 FLEX PLUS [16].

## 2. Materials and Methods

### 2.1. Patients and Sample Collection

A total of 105 patients were enrolled at Chonnam National University Hwasun Hospital in Korea. The male-to-female ratio was approximately 1.84:1. The age distribution ranged from 12 to 84 years old, and the interquartile range was between 57 and 72 years. H-WB, C-WB, and serum samples were collected simultaneously from each patient using a heparin syringe, sodium citrate tube, and serum-separating tube, respectively, and were tested on the same day without being stored. This study was conducted with the approval of the Institutional Review Board of Chonnam National University Hwasun Hospital (CNUHH-2018-096).

### 2.2. Testing Analyzers, Reagents, and Measurements

The ABL90 FLEX PLUS (Radiometer Medical ApS) is a robust benchtop blood gas analyzer that is also available for Cr and BUN measurement [3,4,5]. High precision (<3% of the coefficient of variation (CV) at low and high levels of quality control materials) and linearity (R^2^ > 0.99 at the five levels) were previously validated for Cr and BUN testing using the ABL90 FLEX PLUS by the CLSI guidelines EP05-A3 [17] and EP06-A [18], respectively (Supplementary Table S1 and Supplementary Figure S1). The CLSI EP05 recommends running at least two levels (low and high) of QC materials five times per day for five days, with each run consisting of a minimum of two replicates for each level of QC material. In addition, the linearity evaluation was performed using at least five different concentrations of the Cr and BUN in accordance with CLSI guideline EP06. H-WB is the established primary specimen for the ABL90 FLEX PLUS, according to the manufacturer [5]. For the assessment of the suitability of the candidate specimens, the ABL90 FLEX PLUS was tested using the H-WB (primary specimen), C-WB, and serum samples using the manufacturer’s reagent.

Four automated chemistry analyzers were used, including the ADVIA 1800 (Siemens Healthcare GmbH, Erlangen, Germany), the AU5822 (Beckman Coulter, Brea, CA, USA), the Cobas 8000 c702 (Roche Diagnostics, Basel, Switzerland), and the Hitachi 7600-210 (Hitachi, Tokyo, Japan), to evaluate the performance of the Cr and BUN testing of the ABL90 FLEX PLUS. The external quality assessment program organized by the Korean Association of External Quality Assessment Service assessed the automated chemistry analyzers, especially the Cr measurement, through accuracy-based proficiency testing. For the Hitachi 7600-210, L-Type UN (Wako Pure Chemical Industries, Ltd., Osaka, Japan) and Clinimate^®^ CRE Reagent (SEKISUI MEDICAL CO., Tokyo, Japan) were used for the BUN and Cr measurements, respectively. For the other instruments, reagents exclusive to the manufacturers were used. The Cr and BUN levels were measured in duplicate according to each manufacturer’s instructions. Serum, a standard specimen for chemistry in our institution, was used for the four automated chemistry analyzers.

### 2.3. Data and Statistical Analysis

The means and standard deviations (SDs) of the Cr and BUN levels were calculated for each analyzer and specimen type. A one-way analysis of variance was used to compare the mean values among the different analyzers and specimen types. A *p*-value of less than 0.05 was considered a statistically significant level. For comparison between the test methods or specimen types, each automated chemistry analyzer or H-WB was considered a comparator, respectively.

In accordance with the CLSI guideline EP09C-ED3 for a method comparison study [19], a Bland–Altman plot was visually inspected, and a Passing–Bablok regression was analyzed with a 95% confidence interval (CI) calculated by the bootstrap method using the ‘mcr’ package of R version 1.2.2 [20]. According to the CLSI guideline EP35-ED1 for assessing specimen suitability [16], the equivalence between the primary and candidate specimen types was assessed in terms of systematic difference and imprecision at each medical decision level. The systematic difference at each medical decision level was calculated using the Passing–Bablok regression equation and compared to the total allowable error (TEa) criteria to evaluate the systematic differences between the primary and candidate specimens. The Cr and BUN results of the ABL90 FLEX PLUS were subdivided into three subinterval groups (<0.7, 0.7–1.7, and >1.7 mg/dL for Cr; <20, 20–30, and >30 mg/dL for BUN), which included each medical decision point, to compare the precision of the primary and candidate specimen types. The precision of each specimen type was obtained from the results of two replicates and calculated using the following formula:

(1) $$SD_{specimen\;type}^{2}=\frac{1}{K}\overset{K}{\underset{i=1}{\sum }}\frac{{\left(X_{i,2}-X_{i,1}\right)}^{2}}{2}$$

where *SD_specimen type_* is the average SD of the two replicates of the sample of a specific specimen type, K is the sample number of each specimen type, and *X*_1_ and *X*_2_ are the replicates of the sample. The calculated ratio of *SD_candidate_*/*SD_primary_* (SD ratio) was considered clinically acceptable for precision when the following criteria were satisfied: (i) the SD ratio is less than or equal to 1.00; (ii) the SD ratio is over 1.00, but that of the 95% CI includes 1.00; or (iii) the SD ratio and the lower bound of the 95% CI is over 1.00, but the imprecision for the medical decision level are within the allowable limits [16]. Rounding was performed only on the final results.

The medical decision levels were determined as 0.6, 1.6, and 6.0 mg/dL for Cr, and 6, 26, and 50 mg/dL for BUN [21,22]. The allowable CV (2.3% for Cr; 7.0% for BUN) and TEa criteria (7.4% for Cr; 17.8% for BUN) were set using the desirable specification from the European Federation of Clinical Chemistry and Laboratory Medicine (EFLM) Biological Variation Database [23].

## 3. Results

### 3.1. Comparative Analysis According to Analyzers or Sample Types

**Figure 1:**
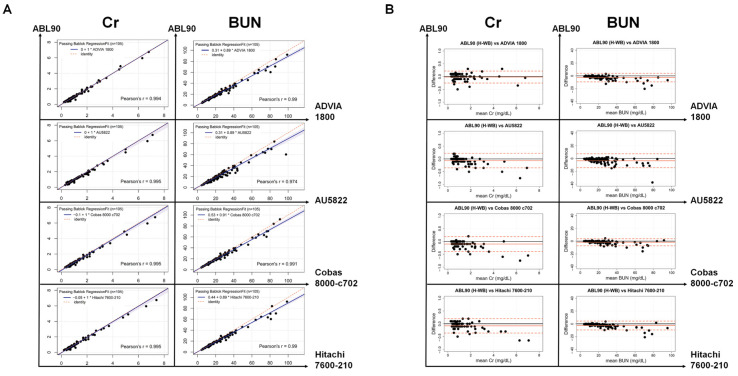
Passing–Bablok regression (**A**) and Bland–Altman plot (**B**) of Cr and BUN testing using the ABL90 FLEX PLUS and H-WB compared with the four other automated chemistry analyzers. (**A**) The blue-colored area represents the 95% CI of the regression line. (**B**) The black and orange solid lines indicate the zero and mean differences, respectively. The dashed orange line represents the two SDs of the mean difference. Cr: creatinine; BUN: blood urea nitrogen; H-WB: heparinized whole blood.

The Cr and BUN results of the ABL90 FLEX PLUS, compared with the four automated chemistry analyzers, are illustrated in Table 1 and Figure 1. The mean values of the Cr and BUN are not statistically different between the ABL90 FLEX PLUS and the other four chemistry analyzers (Table 1A). The correlation coefficients exceeded 0.994 (range: 0.994 to 0.995) and 0.974 (range 0.974 to 0.991) for Cr and BUN, respectively, which indicate high correlations between the ABL90 FLEX PLUS and each automated chemistry analyzer (Figure 1A). The slopes of the Passing–Bablok regression between the ABL90 FLEX PLUS and the four chemistry analyzers were all 1.00 for Cr and ranged from 0.89 to 0.91 for BUN. The intercepts of Cr and BUN ranged from −0.10 to 0.00 and from 0.31 to 0.53, respectively. The mean differences between the ABL90 FLEX PLUS and the ADVIA 1800, AU5822, Cobas 8000 c702, and Hitachi 7600-210 were −0.01, −0.06, −0.10, and −0.08 mg/dL for the Cr testing, and −2.70, −3.51, −1.73, and −2.49 mg/dL for the BUN testing, respectively (Table 1A and Figure 1B).

### 3.2. Assessment of Equivalence between the Primary and Candidate Specimens Using the ABL90 FLEX PLUS

#### 3.2.1. Evaluation of the Systematic Difference among Specimen Types

**Figure 2:**
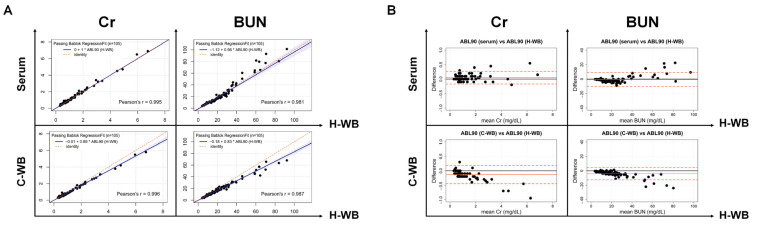
Passing–Bablok regression (**A**) and Bland–Altman plot (**B**) of Cr and BUN testing using the ABL90 FLEX PLUS comparing the primary specimen (H-WB) and candidate specimens (serum and C-WB). (**A**) The blue-colored area represents the 95% CI of the regression line. (**B**) The black and orange solid lines indicate the zero and mean differences, respectively. The dashed orange line represents the two SDs of the mean difference. Cr: creatinine; BUN: blood urea nitrogen; H-WB: heparinized whole blood; C-WB: sodium-citrated whole blood.

There were no significant differences in the mean values of Cr and BUN results among the specimen types using the ABL90 FLEX PLUS (Table 1B). The correlation coefficients of the serum and C-WB compared to that of the H-WB were 0.995 and 0.996 for Cr, and 0.981 and 0.987 for BUN, respectively, using the ABL90 FLEX PLUS (Figure 2A). The slopes of the serum and C-WB compared to that of the H-WB were 1.00 and 0.89 for Cr, and 0.98 and 0.83 for BUN, respectively, in the Passing–Bablok regression analysis (Table 2), and the intercepts of the serum and C-WB compared to that of the H-WB were 0.00 and −0.01 for Cr, and −1.12 and −0.18 for BUN, respectively. The mean difference (% mean difference) in the serum and C-WB compared to the H-WB were 0.04 (4.26%) and −0.14 mg/dL (−12.77%) for Cr, and −0.58 (−9.22%) and −4.00 mg/dL (−18.72%) for BUN, respectively (Table 1B and Figure 2B).

**Figure 3:**
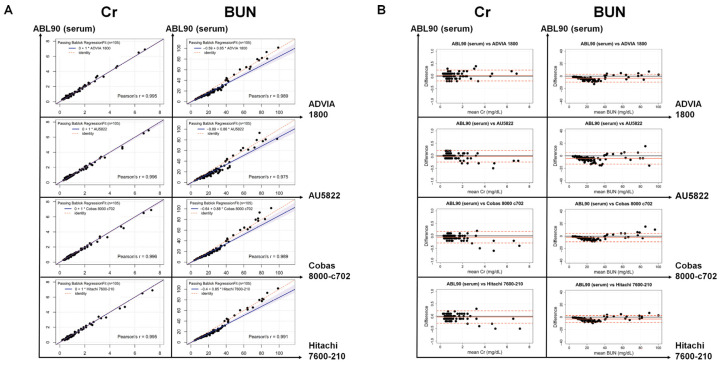
Passing–Bablok regression (**A**) and Bland–Altman plot (**B**) of Cr and BUN testing comparing the four automated chemistry analyzers and the ABL90 FLEX PLUS using serum. (**A**) The blue-colored area represents the 95% CI of the regression line. (**B**) The black and orange solid lines indicate the zero and mean differences, respectively. The dashed orange line represents the two SDs of the mean difference. Cr: creatinine; BUN: blood urea nitrogen.

Systematic differences at each medical decision level were investigated for the Cr and BUN results according to the CLSI EP35-ED1. Compared to the H-WB results using the ABL90 FLEX PLUS for Cr, the systematic difference in the serum was 0% at all medical decision levels, and those of the C-WB were −12.96%, −11.81%, and −11.30% at the 0.6, 1.6, and 6.0 mg/dL medical decision levels, respectively (Table 2). The systematic differences for BUN at the 6, 26, and 50 mg/dL medical decision levels were −21.03%, −6.72%, and −4.66% in the serum, respectively, and −19.87%, −17.57%, and −17.24% in the C-WB, respectively, compared to the H-WB. When the TEa criteria (7.4% for Cr; 17.8% for BUN) were applied, the systematic differences compared to the H-WB were acceptable when a serum specimen was used on the ABL90 FLEX PLUS (except for a low level of BUN testing), whereas the C-WB had inequivalent results at all medical decision levels for both the Cr and BUN testing. In addition, the serum Cr results using the ABL90 FLEX PLUS showed a high correlation (*r* = 0.995 to 0.996) and no systematic difference at all medical decision levels compared to those from each automated chemistry analyzer (Figure 3 and Table 3). For the serum BUN results of the ABL90 FLEX PLUS, the slope of the Passing–Bablok analysis was 0.85 to 0.88 (*r* = 0.975 to 0.991) and the systemic differences ranged from −28.56% to −21.73%, −17.12% to −14.68%, and −15.82% to −13.50% at the 6, 26, and 50 mg/dL medical decision levels, respectively.

#### 3.2.2. Comparison of Precision among Specimen Types

For the subintervals of Cr testing within the ranges of <0.7, 0.7–1.7, and >1.7 mg/dL, the SD_serum_/SD_H-WB_ ratios (95% CI) were 0.14 (0.10 to 0.20), 1.41 (1.09 to 1.84), and 0.68 (0.40 to 1.15), respectively, and the SD_C-WB_/SD_H-WB_ ratios (95% CI) were 0.35 (0.25 to 0.49), 2.00 (1.54 to 2.61), and 0.73 (0.43 to 1.24), respectively (Table 4). The SD ratios of the BUN testing among the specimen types were below 1.00 at all three subintervals (range: 0.05 to 0.51 for SD_serum_/SD_H-WB_; 0.06 to 0.33 for SD_C-WB_/SD_H-WB_). Evaluating the precision in the candidate specimens for Cr, the SD ratios of the candidate specimens were all below 1.00 except for those of the serum (1.41) and C-WB (2.00) at the subinterval range of 0.7–1.7 mg/dL. However, the percent CV of only the serum specimen was 1.89%, and it satisfied the acceptable criteria (<2.3%) at that subinterval.

## 4. Discussion

In this study, Cr and BUN tests by the ABL90 FLEX PLUS showed comparable performance with four automated chemistry analyzers: the ADVIA 1800 (Siemens), the AU5822 (Beckman Coulter), the Cobas 8000 c702 (Roche Diagnostics), and the Hitachi 7600-210 (Hitachi). In addition, an evaluation of equivalence between the blood sample types for Cr and BUN testing of the ABL90 FLEX PLUS in accordance with the latest CLSI guidelines EP35-ED1 showed that only serum was suitable. Given that serum Cr is used for eGFR calculation [15,24,25], these findings provide useful information for interpreting Cr results in situations where an institution is using the ABL90 FLEX PLUS concurrently with one or more chemistry analyzers to measure Cr for the diagnosis and monitoring of renal impairment. To our knowledge, this is the first study to show the equivalence of serum to H-WB, which is the primary recommended sample for Cr and BUN testing by the ABL90 FLEX PLUS, according to the up-to-date CLSI guideline [16].

There have been many studies comparing and evaluating POC devices and one automated chemical device in a central laboratory in various Cr-measuring POC devices, including the ABL90 FLEX PLUS and i-STAT (Abbott) [3,4,6,26,27]. However, each performance of POC Cr testing has varied according to the study cohort or any automated analyzers used for comparison study, making the interpretation of these results difficult [2,3]. To address this issue, 105 paired samples, including H-WB, serum, and C-WB, were used in this study and measured for Cr and BUN in duplicate by four automated chemistry analyzers.

According to the results from accuracy-based proficiency testing of Cr measurements from 2011 to 2017 in Korea [1], the bias found according to the chemistry analyzers was as follows: The Roche and Beckman instruments had a bias close to zero, and the Siemens instrument had a slightly negative bias without statistical significance, while the Hitachi instrument using the Sekisui reagent showed a positive bias over total error. Similarly, our study showed a higher mean value of Cr from the Hitachi 7600-210, and a lower mean value from the ADVIA 1800 among the four chemistry analyzers tested, though there were no statistical differences. The serum Cr levels using the ABL90 FLEX PLUS were in a range between those of the Hitachi 7600-210 and ADVIA 1800 and disclosed no systematic differences among the four automated analyzers at the medical decision levels (Table 3). As a result, in addition to equivalent use with H-WB, the Cr measurements from serum using the ABL90 FLEX PLUS showed results comparable with the other automated chemistry analyzers used worldwide.

The serum specimens showed concordant results with H-WB for the accuracy and precision of the Cr results, whereas the serum BUN level using the ABL90 FLEX PLUS was slightly lower compared to that of the H-WB and even compared to the four chemistry analyzers. These results may also have been influenced by the concentration distribution of the collected samples, considering that negative differences and higher systematic differences were observed mostly at the lower BUN levels (Figure 3A and Table 3). At the low medical decision point (6 mg/dL) for BUN, the systematic differences between the serum and H-WB were unacceptable (Table 2). Hence, a further study involving more patients with higher levels (>40 mg/dL) of BUN may be needed. In addition, if the above finding shows consistency in a future study, careful interpretation of the BUN testing in serum by the ABL90 FLEX PLUS at a lower level and equivalent use with the other automated analyzers may require additional calibration procedures.

WB samples for Cr testing enable a rapid turnaround time with a minimal pre-analytic process. In contrast, serum was originally used for the Modification of Diet in Renal Disease (MDRD) Study equation of eGFR and provides relatively stable results over time [15]. Regarding the imprecision in each specimen compared to H-WB, the primary specimen, the SD_serum_/SD_H-WB_ were all less than 1.00, except for the subinterval of 0.7 to 1.7 mg/dL in the Cr testing. The precision of the WB specimens was similar to that of the serum. Using duplicates of patient WB samples or the WB matrix for Cr testing, a few studies have reported CVs of approximately 3–6% at all concentrations [2,28,29], which is consistent with our findings and exceeds the desirable criteria for imprecision based on biological variability. Moreover, the percent CV of the WB specimens (H-WB and C-WB) was above the allowable criteria only in the subinterval with a low Cr concentration (<0.7 mg/dL). According to the database of biological variability for Cr testing [23], the existing allowable CVs and TEa goals are derived from studies based on serum and plasma. In this context, the accumulation of studies regarding imprecisions based on WB may be helpful for managing Cr testing in POC analyzers. However, C-WB, as another type of WB specimen, consistently showed negative bias compared to H-WB for both Cr and BUN. The negative bias of Cr and BUN testing in C-WB by the ABL90 FLEX PLUS was more prominent when compared to the four other automated analyzers (Supplementary Figure S2). These results may partly explain the bias due to the dilution effect of the anticoagulant, which accounts for one-tenth of the total volume, or the innate negative effects of the citrate solution [30,31]. However, the bias, especially for the Cr results, might be problematic because the eGFR could be overestimated, possibly overlooking some patients with impaired kidney function. In addition, the C-WB showed an unallowable CV (3.17%) and clinically unacceptable SD ratios (2.00 with 95% CI of 1.54 to 2.61) compared to the H-WB at the subinterval range of 0.7–1.7 mg/dL for Cr testing using the ABL90 FLEX PLUS. This result is notable because the range of 0.7–1.7 mg/dL in the Cr testing included an eGFR level of 60 mL/min/1.73 m^2^, considering the age, gender, and race of the patient [15]. Consequently, C-WB is not recommended for Cr and BUN testing using the ABL90 FLEX PLUS.

## 5. Conclusions

In conclusion, the Cr testing in serum and H-WB by the ABL90 FLEX PLUS was comparable with that by four automated chemistry analyzers commonly used in clinical laboratories. Therefore, they can be equivalently used in situations where the ABL90 FLEX PLUS is used simultaneously with one or more chemistry analyzers in one institute. However, C-WB is not recommended.

## Figures and Tables

**Table 1:** The summary of the Cr and BUN results using the ABL90 FLEX PLUS compared to the four automated chemistry analyzers (A) and specimen types (B).

**(A) Cr and BUN Results according to Analyzers**						
	**Cr**			**BUN**		
	**Mean (SD), mg/dL**	**Mean Difference ^1^ (95% CI), mg/dL**	***p*-Value ^2^**	**Mean (SD), mg/dL**	**Mean Difference ^1^ (95% CI), mg/dL**	***p*-Value ^2^**
ABL90 FLEX PLUS	1.15 (1.08)	-	0.97	22.72 (17.11)	-	0.73
ADVIA 1800	1.17 (1.09)	−0.01 (−0.04, 0.01)	0.97	25.41 (19.29)	−2.70 (−3.34, −2.05)	0.73
AU5822	1.21 (1.17)	−0.06 (−0.09, −0.04)	0.97	26.23 (20.53)	−3.51 (−4.57, −2.46)	0.73
Cobas 8000 c702	1.25 (1.17)	−0.10 (−0.12, −0.07)	0.97	24.45 (18.52)	−1.73 (−2.26, −1.20)	0.73
Hitachi 7600-210	1.23 (1.17)	−0.08 (−0.10, −0.05)	0.97	25.21 (19.45)	−2.49 (−3.16, −1.82)	0.73
**(B) Cr and BUN results according to specimen type on ABL90 FELX PLUS**						
	**Cr**			**BUN**		
	**Mean (SD), mg/dL**	**Mean Difference ^1^ (95% CI), mg/dL**	***p*-Value ^2^**	**Mean (SD), mg/dL**	**Mean Difference ^1^ (95% CI), mg/dL**	***p*-Value ^2^**
H-WB	1.15 (1.08)	-	0.42	22.72 (17.11)	-	0.19
Serum	1.19 (1.11)	0.04 (0.02, 0.06)	0.42	22.13 (20.29)	−0.58 (−1.52, 0.35)	0.19
C-WB	1.01 (0.95)	**−0.14 (−0.17, −0.11)**	0.42	18.71 (13.58)	**−4.00 (−4.84, −3.16)**	0.19

Highest absolute mean differences among analyzers and specimen types are marked in bold. ^1^ The results of four automated analyzers and H-WB were considered the comparator for the comparison among analyzers and specimen types, respectively. ^2^ Calculated by a one-way analysis of variance to compare the mean of the Cr and BUN testing among the different analyzers or specimen types. Cr: creatinine; BUN: blood urea nitrogen; H-WB: heparinized whole blood; C-WB: sodium-citrated whole blood; SD: standard deviation; CI: confidence interval.

**Table 2:** Passing–Bablok regression and systematic differences between the primary specimen (H-WB) and the candidate specimen types for Cr and BUN testing using the ABL90 FLEX PLUS.

Analyte	Candidate Specimen Type	Regression Equation		Correlation Coefficient (*r*) ^2^	Systematic Difference of Analytes (95% CI ^1^), mg/dL, at the Medical Decision Level ^3^ of:			Systematic Difference of Analytes ^4^ (95% CI ^1^) at the Medical Decision Level ^3^ of:		
		Slope (95% CI ^1^)	Intercept (95% CI ^1^)		Low	Medium	High	Low	Medium	High
Cr	Serum	1.00 (1.00, 1.00)	0 (0, 0)	0.995	0 (0, 0)	0 (0, 0)	0 (0, 0)	0 (0, 0)	0 (0, 0)	0 (0, 0)
	C-WB	0.89 (0.86, 0.93)	−0.01 (−0.05, 0.02)	0.996	−0.08 (−0.09, −0.06)	−0.19 (−0.22, −0.16)	−0.68 (−0.84, −0.48)	−12.96 (−15.32, −10.00)	−11.81 (−13.39, −10.10)	−11.30 (−14.05, −8.33)
BUN	Serum	0.98 (0.89, 1.06)	−1.12 (−2.35, −0.14)	0.981	−1.26 (−2.09, −0.74)	−1.75 (−2.84, −0.36)	−2.33 (−5.25, 1.12)	−21.03 (−34.84, −12.35)	−6.72 (−10.94, −1.37)	−4.66 (−10.51, 2.25)
	C-WB	0.83 (0.79, 0.87)	−0.18 (−0.75, 0.58)	0.987	−1.19 (−1.52, −0.65)	−4.57 (−5.20, −3.88)	−8.62 (−10.17, −7.05)	−19.87 (−25.38, −10.90)	−17.57 (−19.99, −14.93)	−17.24 (−20.34, −14.10)

^1^ Bootstrap confidence interval (1000 iterations). ^2^ Calculated using the Pearson correlation coefficient. ^3^ Respective medical decision levels (low, medium, and high) were 0.6, 1.6, and 6.0 mg/dL for Cr and 6, 26, and 50 mg/dL for BUN. ^4^ Rounding was performed only on the final results of systematic difference with 95% CI. H-WB: heparinized whole blood; Cr: creatinine; BUN: blood urea nitrogen; C-WB: sodium-citrated whole blood; CI: confidence interval.

**Table 3:** Passing–Bablok regression and systematic differences between the four automated chemistry analyzers and the ABL90 FLEX PLUS using serum for Cr and BUN testing.

Analyte	Comparator Analyzers	Regression Equation		Correlation Coefficient (*r*) ^2^	Systematic Difference of Analytes (95% CI ^1^), mg/dL, at the Medical Decision Level ^3^ of:			Systematic Difference of Analytes ^4^ (95% CI ^1^) at the Medical Decision Level ^3^ of:		
		Slope (95% CI ^1^)	Intercept (95% CI ^1^)		Low	Medium	High	Low	Medium	High
Cr	ADVIA 1800	1.00 (1.00, 1.00)	0 (0, 0)	0.995	0 (0, 0)	0 (0, 0)	0 (0, 0)	0 (0, 0)	0 (0, 0)	0 (0, 0)
	AU5822	1.00 (0.95, 1.00)	0 (0, 0.03)	0.996	0 (0, 0)	0 (−0.04, 0)	0 (−0.25, 0)	0 (0, 0.65)	0 (−2.68, 0)	0 (−4.09, 0)
	Cobas 8000 c702	1.00 (0.97, 1.00)	0 (−0.10, 0.01)	0.996	0 (−0.10, 0)	0 (−0.10, 0)	0 (−0.18, 0)	0 (−16.67, 0)	0 (−6.25, 0)	0 (−2.92, 0)
	Hitachi 7600-210	1.00 (1.00, 1.00)	0 (0, 0)	0.995	0 (0, 0)	0 (−0.01, 0)	0 (−0.09, 0)	0 (0, 0)	0 (−0.93, 0)	0 (−1.45, 0)
BUN	ADVIA 1800	0.85 (0.79, 0.94)	−0.59 (−2.02, 0.26)	0.989	−1.47 (−2.45, −0.96)	−4.39 (−5.01, −3.37)	−7.89 (−10.46, −4.69)	−24.46 (−40.81, −15.98)	−16.87 (−20.41, −12.97)	−15.77 (−20.91, −9.37)
	AU5822	0.86 (0.80, 0.94)	−0.89 (−2.19, 0.05)	0.975	−1.71 (−2.56, −1.16)	−4.45 (−5.42, −3.60)	−7.73 (−10.32, −5.13)	−28.56 (−42.60, −19.33)	−17.12 (−20.84, −13.83)	−15.47 (−20.64, −10.26)
	Cobas 8000 c702	0.88 (0.81, 0.95)	−0.64 (−1.65, 0.34)	0.989	−1.37 (−2.01, −0.73)	−3.82 (−4.56, −2.80)	−6.75 (−9.02, −4.05)	−22.87 (−33.43, −12.22)	−14.68 (−17.56, −10.78)	−13.50 (−18.05, −8.09)
	Hitachi 7600-210	0.85 (0.80, 0.93)	−0.40 (−1.73, 0.34)	0.991	−1.30 (−2.20, −0.86)	−4.31 (−5.04, −3.33)	−7.91 (−9.97, −5.06)	−21.73 (−36.75, −14.34)	−16.56 (−19.39, −12.81)	−15.82 (−19.94, −10.12)

^1^ Bootstrap confidence interval (1000 iterations). ^2^ Calculated using the Pearson correlation coefficient. ^3^ Respective medical decision levels (low, medium, and high) were 0.6, 1.6, and 6.0 mg/dL for Cr and 6, 26, and 50 mg/dL for BUN. ^4^ Rounding was performed only on the final results of systematic difference with 95% CI. Cr: creatinine; BUN: blood urea nitrogen; CI: confidence interval.

**Table 4:** Evaluation of precision for equivalence between the primary and candidate specimens in Cr (A) and BUN (B) testing using the ABL90 FLEX PLUS.

**(A) SD ratios between primary and candidate specimen for Cr**						
**Range of the Subintervals (No. of Subjects)**		**H-WB (Primary) ^1^**	**Serum**	**C-WB**	**SD_serum_/SD_H-WB_ (95% CI) ^2^**	**SD_C-WB_/SD_H-WB_ (95% CI) ^2^**
<0.7 mg/dL (34)	Mean	0.51	0.56	0.47		
	SD	0.08	0.01	0.03	0.14 (0.10, 0.20)	0.35 (0.25, 0.49)
	%CV	**16.54**	2.15	**6.35**		
0.7–1.7 mg/dL (56)	Mean	0.98	1.00	0.84		
	SD	0.01	0.02	0.03	**1.41 (1.09, 1.84)**	**2.00 (1.54, 2.61)**
	%CV	1.36	1.89	3.17		
>1.7 mg/dL (15)	Mean	3.28	3.34	2.86		
	SD	0.07	0.04	0.05	0.68 (0.40, 1.15)	0.73 (0.43, 1.24)
	%CV	2.01	1.34	1.69		
Total (105)	Mean	1.15	1.19	1.01		
	SD	0.06	0.02	0.03	0.41 (0.34, 0.50)	0.57 (0.47, 0.69)
	%CV	4.80	1.92	3.13		
**(B) SD ratios between primary and candidate specimens for BUN**						
**Range of the Subintervals (No. of Subjects)**		**H-WB (Primary) ^1^**	**Serum**	**C-WB**	**SD_serum_/SD_H-WB_ (95% CI) ^2^**	**SD_C-WB_/SD_H-WB_ (95% CI) ^2^**
<20 mg/dL (62)	Mean	12.71	11.15	10.66		
	SD	1.04	0.06	0.07	0.05 (0.04, 0.07)	0.06 (0.05, 0.08)
	%CV	8.18	0.51	0.62		
20–30 mg/dL (22)	Mean	24.36	21.02	20.42		
	SD	0.40	0.15	0.10	0.36 (0.24, 0.56)	0.24 (0.15, 0.36)
	%CV	1.66	0.70	0.47		
>30 mg/dL (21)	Mean	50.52	55.71	40.71		
	SD	1.28	0.65	0.42	0.51 (0.33, 0.79)	0.33 (0.21, 0.51)
	%CV	2.53	1.17	1.03		
Total (105)	Mean	22.72	22.13	18.71		
	SD	1.00	0.30	0.20	0.30 (0.25, 0.37)	0.20 (0.16, 0.24)
	%CV	4.40	1.37	1.06		

Imprecision results, which exceed the allowable CV and SD ratios of 1.00, are marked in bold. ^1^ H-WB was used as the primary specimen for the Cr and BUN testing using the ABL90 FLEX PLUS according to the manufacturer’s instruction. ^2^ Rounding was performed only on the final results of precision ratios with 95% CI. Cr: creatinine; BUN: blood urea nitrogen; SD: standard deviation; CV: coefficient of variation; H-WB: heparinized whole blood; C-WB: sodium-citrated whole blood; CI: confidence interval.

## Data Availability

Data are available upon appropriate request.

## Supplementary Materials

The following supporting information can be downloaded at: https://www.mdpi.com/article/10.3390/jcm12051940/s1, Figure S1: Linear regression analysis of the expected concentration of Cr (A) or BUN (B) versus the measured concentration by the ABL90 FLEX PLUS; Figure S2: Passing–Bablok regression (A) and Bland–Altman plot (B) of Cr and BUN testing between the four automated chemistry analyzers and the ABL90 FLEX PLUS using C-WB; Table S1: Precision of Cr and BUN measurements using the ABL90 FLEX PLUS analyzer using quality control materials.
