# Supplementary material for: Evaluation of the Accuracy of Cr and BUN Using the ABL90 FLEX PLUS Blood Gas Analyzer and the Equivalence of Candidate Specimens for Assessment of Renal Function
Source: J Clin Med. 2023 Mar 1;12(5):1940. doi: 10.3390/jcm12051940 (PMC10003958; doi:10.3390/jcm12051940)
Supplement: Supplementary file 1 [file jcm-12-01940-s001.zip › jcm-2193491-supplementary.pdf]

Supplemental Figures

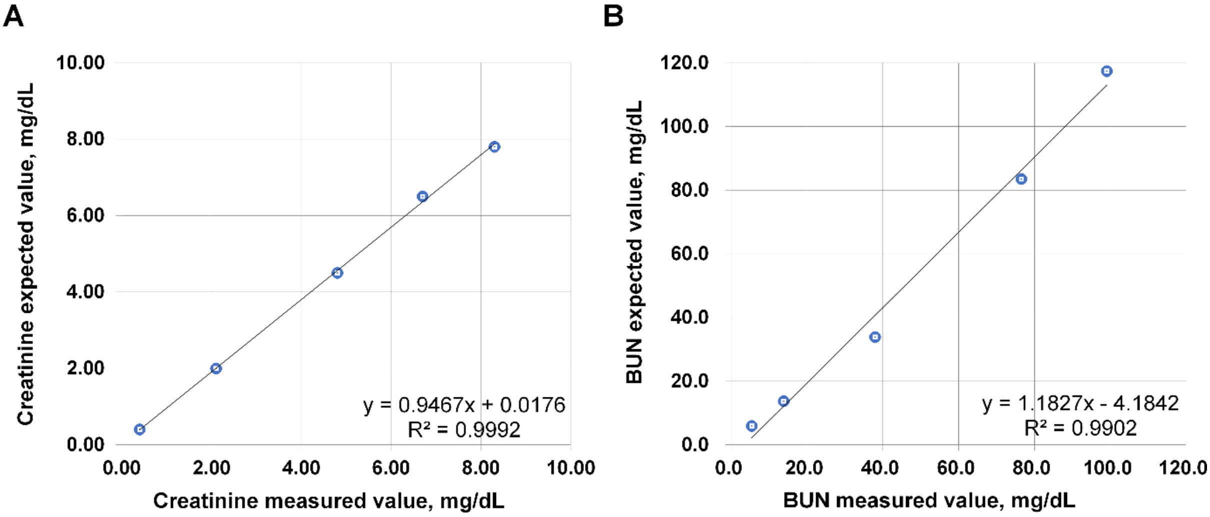

Supplemental Figure S1. Linear regression analysis of the expected concentration of Cr (A) or BUN (B) versus measured concentration by ABL90 FLEX PLUS.

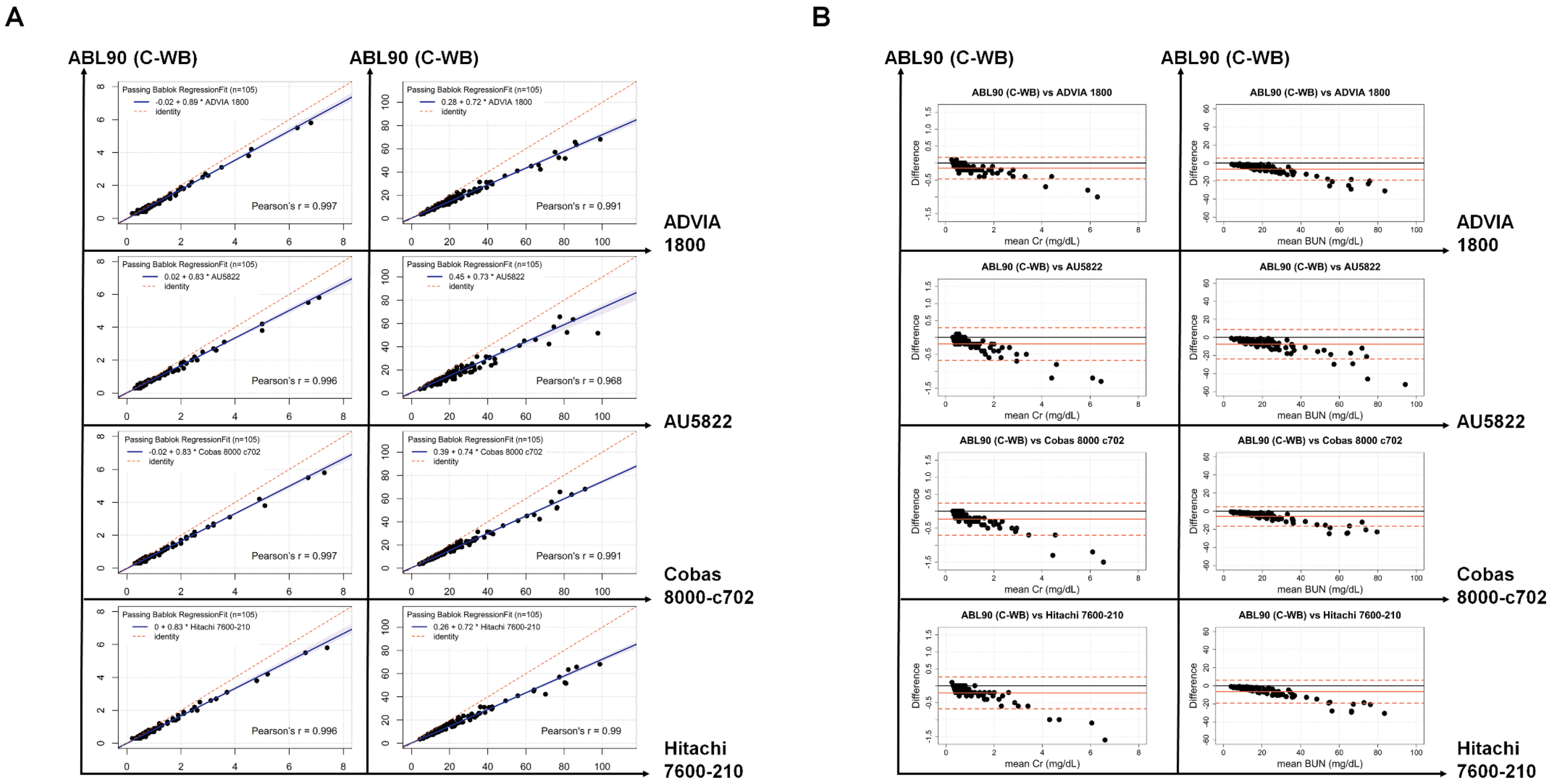

Supplemental Figure S2. Passing-Bablok regression (A) and Bland-Altman plot (B) of Cr and BUN testing between the four automated chemistry analyzers and ABL90 FLEX PLUS using C-WB. A: Blue-colored area represents the 95% CI of regression line. B: The black and

orange solid lines indicates the zero and mean of difference, respectively. The dashed orange line represents two SDs of mean difference. Cr: creatinine; BUN: blood urea nitrogen; C-WB: sodium-citrated whole blood.

Supplemental Table

Supplemental Table S1. Precision of Cr and BUN measurement using ABL90 FLEX PLUS analyzer using quality control materials

Analyte	Level	Mean, mg/dL	Total SD, mg/dL	Total CV, %
Cr	Low	1.40	0.020	1.42
	High	9.50	0.058	0.61
BUN	Low	17.44	0.337	1.93
	High	44.55	1.169	2.62

Cr: creatinine; BUN: blood urea nitrogen; SD: standard deviation; CV: coefficient of variation.
